# The Host Response to a Clinical MDR Mycobacterial Strain Cultured in a Detergent-Free Environment: A Global Transcriptomics Approach

**DOI:** 10.1371/journal.pone.0153079

**Published:** 2016-04-07

**Authors:** Gina Leisching, Ray-Dean Pietersen, Vuyiseka Mpongoshe, Carel van Heerden, Paul van Helden, Ian Wiid, Bienyameen Baker

**Affiliations:** 1 SA MRC Centre for TB Research, DST-NRF Centre of Excellence for Biomedical Tuberculosis Research, Division of Molecular Biology and Human Genetics, Faculty of Medicine and Health Sciences, Stellenbosch University, Stellenbosch, South Africa; 2 Central Analytical Facility (CAF), DNA sequencing unit, Stellenbosch University, Stellenbosch, South Africa; Bose Institute, INDIA

## Abstract

During *Mycobacterium tuberculosis* (*M*.*tb)* infection, the initial interactions between the pathogen and the host cell determines internalization and innate immune response events. It is established that detergents such as Tween alter the mycobacterial cell wall and solubilize various lipids and proteins. The implication of this is significant since induced changes on the cell wall affect macrophage uptake and the immune response to *M*.*tb*. Importantly, during transmission between hosts, aerosolized *M*.*tb* enters the host in its native form, i.e. in a detergent-free environment, thus *in vitro* and *in vivo* studies should mimic this as closely as possible. To this end, we have optimized a procedure for growing and processing detergent-free *M*.*tb* and assessed the response of murine macrophages (BMDM) infected with multi drug-resistant *M*.*tb* (R179 Beijing 220 clinical isolate) using RNAseq. We compared the effects of the host response to *M*.*tb* cultured under standard laboratory conditions (Tween 80 containing medium -R179T), or in detergent-free medium (R179NT). RNAseq comparisons reveal 2651 differentially expressed genes in BMDMs infected with R179T *M*.*tb* vs. BMDMs infected with R179NT *M*.*tb*. A range of differentially expressed genes involved in BMDM receptor interaction with *M*.*tb* (*Mrc1*, *Ifngr1*, *Tlr9*, *Fpr1* and *Itgax*) and pro-inflammatory cytokines/chemokines (*Il6*, *Il1b*, *Tnf*, *Ccl5* and *Cxcl14*) were selected for analysis through qPCR. BMDMs infected with R179NT stimulate a robust inflammatory response. Interestingly, R179NT *M*.*tb* induce transcription of *Fpr1*, a receptor which detects bacterial formyl peptides and initiates a myriad of immune responses. Additionally we show that the host components *Cxcl14*, with an unknown role in *M*.*tb* infection, and Tlr9, an emerging role player, are only stimulated by infection with R179NT *M*.*tb*. Taken together, our results suggest that the host response differs significantly in response to Tween 80 cultured *M*.*tb* and should therefore not be used in infection experiments.

## Introduction

*Mycobacterium tuberculosis* (*M*.*tb*) displays remarkable versatility with its ability to infect mammalian cells and evade host destruction. Specific host cell receptors are required for entry, and the internalization of *M*.*tb* is facilitated by a complex signalling cascade initiated by the host cell upon receptor-ligand binding [1, 2]. The host response to infection has been well documented by a number of *in vitro* and *in vivo* studies which rely on the use of detergents such as Tween to ensure the generation of manageable, non-aggregating cultures. It has been established that Tween-induced changes on the mycobacterial cell wall affect macrophage uptake and the immune response to *M*.*tb* [3, 4]. It is important to note however, that during transmission between hosts, aerosolized *M*.*tb* enters in its native form, i.e. in a detergent-free environment therefore *in vitro* infection experiments should try mimic this as closely as possible.

The inclusion of Tween in growth media was introduced almost 70 years ago [5] and is an efficient and successful approach for obtaining homogenous, non-aggregating cultures. The characteristic “clumping” of *M*.*tb* is attributed to a variety of factors associated with components of the cell envelope, including cell wall lipids such as trehalose dimycolates (TDM) and the HbhA and PE-PGRS proteins [6, 7]. TDMs have a variety of immunostimulatory properties [8, 9] and are vital in nonspecific resistance to infectious agents [10, 11], *in vitro* and *in vivo* responses to infection [12, 13] and play multiple roles in pathogenesis [14]. In addition, literature suggests that the use of Tween compounds alter phenotypic and biochemical characteristics of *M*.*tb* [3, 15–20]. These facts still remain overlooked, and may be that an effective technique for culturing *M*.*tb* without detergent has not been developed. We address this issue by including an optimized protocol for culturing *M*.*tb* without Tween. A recent study has assessed the transcriptome profile of bovine alveolar macrophages after infection with *M*. *bovis* cultured without Tween 80 detergent [21]. Here, the authors present complex patterns of gene regulation which may provide insight into mechanisms used by M. bovis to evade destruction. Here we present the first study to provide the host transcription profile using RNAseq in response to infection with *M*. *tuberculosis* in its native state (i.e. cultured in a detergent-free environment) and provide evidence of a largely differential host response.

## Materials and Methods

### Cells and culture medium

Bone marrow (precursor) cells were isolated from femurs of 6–8 week-old C57Bl/6 female mice as described previously [22] and diluted in RPMI-1640 (containing L-glutamine and Na-bicarbonate; Sigma, USA) supplemented with 10% FBS (Biochrom, Germany) and 10% L-cell conditioned medium (source of CSF-1), as growth medium. Cells were seeded into 6-well tissue culture dishes (Nunc, Thermo Scientific, USA) at 5 x 10 ^5^ cells per well. Precursor cells were allowed 4–5 days to adhere and differentiate into macrophages before washing away undifferentiated cells and refreshing the medium. Growth medium was replaced every second day. Bacterial infection occurred on day 7.

### Growing of detergent-free mycobacteria for infection experiments

Middlebrook7H9 medium (Difco, Becton Dickinson, USA) supplemented with 10% oleic acid-albumin-dextrose-catalase (OADC, Becton Dickinson, USA) and 0.5% glycerol (Merck Millipore, Germany) (no Tween 80/detergent) was prepared. A stock vial of *M*.*tb* that was previously grown in the presence of Tween 80 was used in order to start with little to no clumps and minimize clumping in the starter culture. The bacteria was thawed and then passed 10x through a G25 needle before seeding. Two 10 ml cultures were started in T25 flasks from one stock vial with detergent-free 7H9 medium. The starter culture was grown to an OD_600_ of 0.2–0.3. Each flask was sub-cultured into 5 T25 flasks (10 flasks in total), where 1ml starter culture was diluted in 9 ml detergent-free 7H9 medium and grown to an OD_600_ of 0.3–0.4.

Each flask was split into 2 x T25 flasks where 5 ml culture was added to 5 ml Tween-less 7H9 medium (20 flasks in total) and grown to an OD_600_ of 0.4 to minimize clumping (cultures grown past this OD were observed to clump exponentially, consequently resulting in a significantly lower yield of single-celled bacteria, see S1 Fig). For stocks, all cultures were combined into 4 x 50 ml tubes where after major bacterial clumps were given 10 min to settle out (except BCG which settled out completely in 5 min and therefore given 2 min settling time only). The top 45 ml from each tube was placed into new 50 ml tubes, centrifuged at 460 x g for 5 min and the supernatant discarded. Each pellet was resuspended in 5 ml detergent-free 7H9 medium before combining (20 ml in total) and allowed to stand for a further 10 min to settle out major clumps. The top 17 ml was carefully removed and placed into a new tube and aliquoted either directly or after mixing with glycerol. One millilitre aliquots were frozen at -80°C for future infection experiments.

### Preparing detergent-free mycobacteria for infection—the syringe-settle-filtrate (ssf) method

Stock vials were thawed and clumps were disrupted by passing through a 1 ml tip 10 times followed by syringing [23] 10 times (20 passes) through a G25 needle. Major clumps were allowed up to 10 min to settle [24], where after the top 750 μl was added to 4.25 ml cellular growth medium (in this case RPMI 1640 with 10% G-CSF). The 5 ml bacterial suspension was filtered immediately through a 5.0 μm pore size filter [4] (Merck Millipore, Germany) and 10% FBS added. The required volume (depending on titration and MOI) was then added to bone marrow derived macrophages (BMDMs) in complete medium. This method (**s**yringing, **s**ettling and **f**iltration, SSF) was also used for titrating *M*.*tb* stocks (no FBS added in this case), whereby 3 stock vials were processed to obtain an average CFU. The SSF method (as developed by the authors) was accepted as currently the best way to produce single mycobacteria from detergent-free grown cultures (S1 Fig). This protocol may also be applied to fast-growing mycobacteria and extrapolated to *in vivo* aerosolization studies.

### Bacterial strain and infection conditions

*Mycobacterium tuberculosis* R179 (Beijing 220 clinical isolate, RIF, EMB, PZA resistance with low-level INH resistance) [25, 26] was used for infection and cultured in Tween containing 7H9 (supplemented with 10% OADC, 0.5% glycerol and 0.05% Tween 80—referred to as R179T) and Tweenless/detergent-free (referred to as R179NT) 7H9 medium (supplemented with 10% OADC, 0.5% glycerol). Prior to infection, R179T bacteria was processed in the same way (described above) as R179NT bacteria to control for the processing method. BMDMs were infected with either R179T or R179NT at MOI 1–3 and allowed 4 h for uptake. S3 Fig indicates percentage uptake of detergent-free *M*.*tb*, as well as evidence of BMDMs internalizing single-celled M.tb. The cells were then washed 3 times with phosphate buffered saline (PBS), and incubated for an additional 8 hours in complete medium (12 h in total). Uninfected BMDMs served as the control.

### RNA extraction and mRNA enrichment

BMDM RNA was extracted using the RNeasy® Plus Mini Kit (Cat. No. 74134, Qiagen, Limburg, Netherlands) according to the manufacturer’s instructions immediately after the infection period. RNA quality and quantity was assessed using the Agilent 2100 Bioanalyser. RNA samples with RNA integrity Number (RIN) above 9.0 were used for RNAseq and qPCR. Two rounds of mRNA enrichment wereperformed using the Dynabeads® mRNA DIRECT™ Kit (Cat. No. 61012, Ambion, Life Technologies, Oslo, Norway) according to the manufacturer’s instructions. mRNA was frozen immediately at -80°C until RNAseq was performed. Three biological replicates for RNA-seq (each biological replicate run in triplicate) and qPCR were used (each biological replicate run in duplicate).

### RNA-seq

A barcoded RNA library was constructed for each of the 3 biological replicates in triplicate using the AB Library Builder™ Whole Transcriptome Core Kit for 5500 Genetic Analysis Systems (Cat. No. 4472690, Applied Biosystems, Life Technologies). The concentrations of the libraries were normalized using qPCR. To prevent any potential bias being introduced during emulsion PCR or sequencing, the nine libraries were mixed prior to emulsion PCR using two E120 modules and the SOLiD® EZ Bead™ System (Cat. No. 4448419, Applied Biosystems, Life Technologies). After enrichment the libraries were loaded onto two flow cells for sequencing. Paired-end sequencing (75/35 bp) was performed on a SOLiD™5500xl. The run was continuously monitored for data quality using the standard tools in the Instrument Control software.

Analysis was performed using LifeScope 2.5 (http://www.lifetechnologies.com/lifescope) and Partek Flow Software (Partek Inc., St Louis, MO, USA, build 4.0.15). The reads were mapped to version GRCm38/*mm10* of the mouse reference genome using LifeScope software. The total number of reads mapped by LifeScope software was extracted from the BAMSTATS output, along with the number of unmapped reads and reads with a mapQV of less than 10. The mapped reads were exported as.bam files which could be imported into Partek Flow software. The post-alignment QC module of Partek Flow was used to visualize the average base quality score per position as well as the mapping quality per alignment. The mapped reads were quantified using the RefSeq transcripts-2015-02-02 annotation for quantification using the Partek E/M method. Strict paired-end compatibility was enforced as well as a requirement for junction reads to match defined annotated introns.

### Analysis of gene expression level

The mean expression values were calculated for each gene for the various biological repeats. Differential gene expression was done using Partek Flow Software. In short, the gene count data was normalized using FPKM (Partek performs RPKM using both reads in a pair, but still refers to it as RPKM and not FPKM) and the Gene Specific Analysis (GSA) algorithm was used to identify potential differentially expressed genes (Partek Settings used: S1 methods). Only regions with a minimum coverage of at least one were considered and false discovery rates (FDR) were also calculated. The data was filtered to remove gene with low expression levels and FDR of more than 0.05. Only fold changes of ≥ 2 or ≤ −2 were considered for hierarchical clustering. Both the samples and the genes were clustered. Canonical pathway analysis was performed using Ingenuity Pathway Analysis (IPA, http://www.ingenuity.com). Canonical pathways analysis identified curated pathways from the IPA Knowledge Base that were significantly associated with the dataset.

### Quantitative qPCR

For cDNA synthesis, 0.5 μg RNA was converted to cDNA using the Quantitect® Reverse Transcription Kit (Cat. No. 205311, Qiagen, Limburg, Netherlands). qPCR amplification was performed in 96-well plates and run on a LightCycler® 96 system (Roche, Germany). LightCycler® 480 SYBR Green I Master (Cat. No. 04887352001, Roche, Germany) was used with the following QuantiTect® primer assays (Qiagen, Limburg, Netherlands) at a reaction volume of 20 μl: *Ifngr1* (Mm_Ifngr1_1_SG, Cat. No. QT00092582), *Fpr1* (Mm_Fpr1_1_SG, Cat. No. QT00258139), *Itgax* (Mm_Itgax_1_SG, Cat. No. QT00113715), *TLR9* (Mm_Tlr9_2_SG, Cat. No. QT01043049), *Mrc1* (Mm_Mrc1_1_SG, Cat. No. QT00103012), *Ccl5* (Mm_Ccl5_2_SG, Cat. No. QT01747165), *Cxcl14* (Mm_Cxcl14_1_SG, Cat. No. QT00171157), *Il6* (Mm_Il6_1_SG, Cat. No. QT00098875), *Tnf* (Mm_Tnf_1_SG.Cat. No. QT00104006), *Il1b* (Mm_Il1b_2_SG, Cat. No. QT01048355). Reference genes used were *Lamp2* (Mm_Lamp2_1_SG, Cat. No. QT00101059), *Ubc* (Mm_Ubc_1_SG, Cat. No. QT00245189), *B2m* (Mm_B2m_2_SG, Cat. No. QT01149547) and *G6pd* (Mm_G6pdx_1_SG, Cat. No. QT00120750). These reference genes were chosen according to stable expression levels from RNAseq data and confirmed through qPCR. The amplification procedure entailed 45 cycles of 95°C for 10 s followed by 60°C for 10s and finally 72°C for 10s. Relative expression analysis was performed using the equation N = N_0_ x 2^Cp^ (LightCycler®96 software, Roche), normalizing against the above mentioned reference genes. The Pearson correlation (*r*) between qPCR and RNAseq gene-expression fold-change was estimated. All samples were run in triplicate with a positive control and a non-reverse transcription control in accordance with the MIQE guidelines

### Animal housing and ethics statement

Animals were housed 3 per cage in a temperature-controlled room with a 12-h light-dark cycle and had free access to food and water. This research study was approved by the Stellenbosch University Animal Ethics committee on Animal Care and Use and complies with the South African Animal Protection Act (Act no 71, 1962). Animal Ethics No. SU-ACUD14-00041.

### Statistical analysis

Statistical significance was performed with GraphPad Prism software. ANOVA was used for comparisons involving 3 or more groups. All values expressed as means ± SEM with a p < 0.05 considered as significant.

## Results

Since our study is based on *M*.*tb* cultured without the use of detergent, we have included in this paper an optimized protocol for growing and preparing detergent-free *M*.*tb* for use in infection experiments. Although parts of this protocol have been published previously [4, 23, 24], the final yield is too low for use in infection experiments. We have carefully combined and optimised these procedures, as well as added in additional steps in order to circumvent this issue. We attribute thesuccess of this method to a combination of processing steps. Firstly, cultures were grown up to an OD of 0.4 in multiple standing flasks (as opposed to one roller bottle/shaking flask), secondly, prior to infection cultures were syringed to break up larger bacterial aggregates. Thirdly, a settling time was introduced to allow the remaining aggregates to settle out, and lastly filtration was applied in 5 ml of the host-cell growth medium, which was the final step in obtaining single-celled mycobacteria (S1 and S2 Figs).

### BMDMs infected with R179-Tween and R179 non-Tween *M*.*tb* exhibit different transcriptome profiles

**Fig 1:**
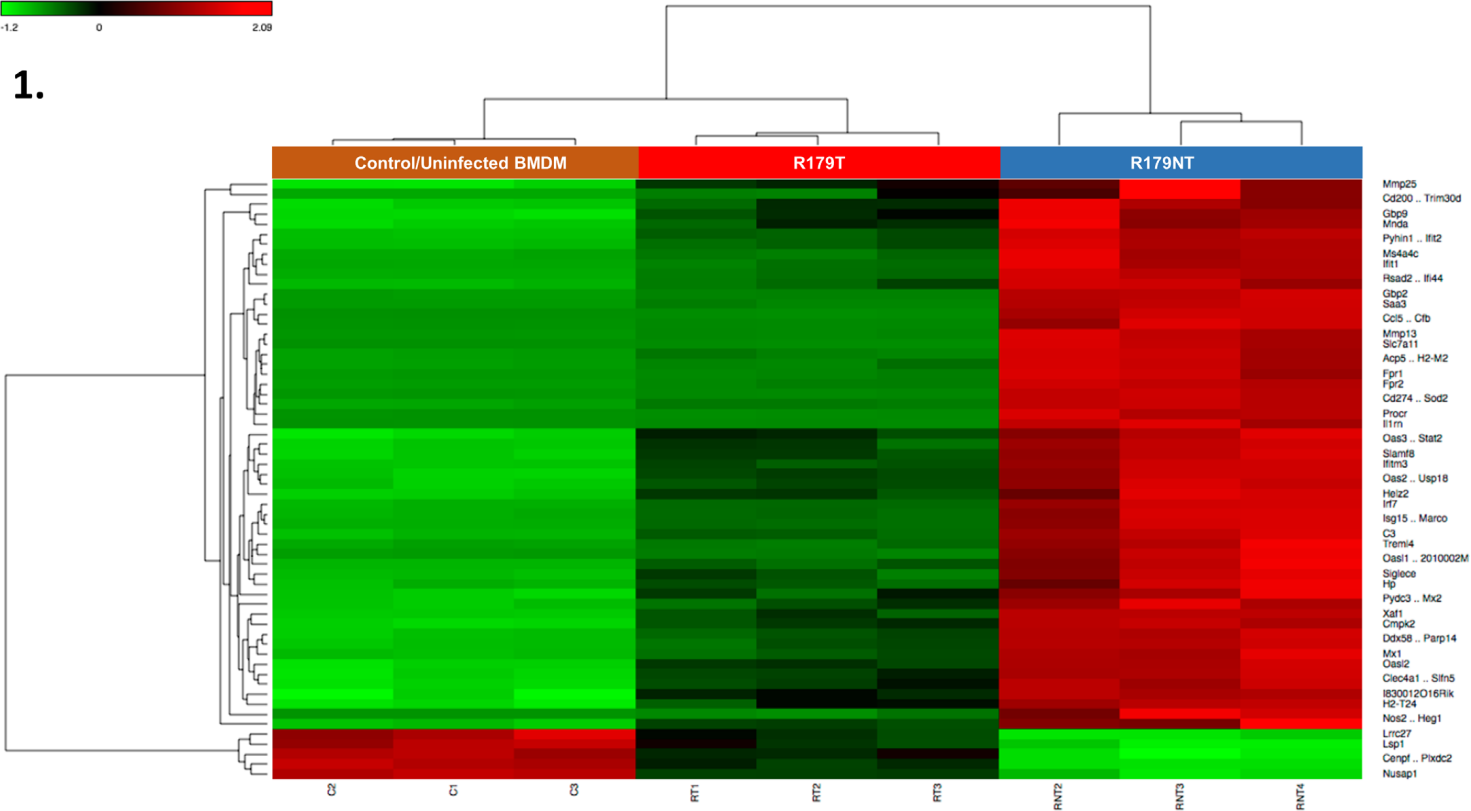
Differential expression of host gene transcripts in R179T versus R179NT infected BMDMs. Heatmap visualization of differentially expressed transcripts as analyzed by RNA-seq where R179T and R179NT were compared to uninfected BMDMs. Transcripts with significant fold changes, based on both fold change and FDR adjusted P-value threshold, are shown in the heat map. The level of expression of each gene, in each sample, relative to the mean level of expression of that gene across all of the samples, is represented by using a red–green color scale as shown in the key with a range of − 1.2 to + 2.09 on a log (10) scale. Gene names are indicated to the right of the heat map and bacterial growth conditions are shown at the top. Red = upregulation, green = downregulation. Dendrogram indicates sample clustering. Differentially expressed genes defined as having an FC >2.0 and FDR <0.05. Analysis was conducted on three biological replicates (C1, 2, 3, RT1, 2, 3 and RNT1, 2, 3). BMDMs infected with detergent-free *M*.*tb* exhibit a differential infection profile. C—control/uninfected BMDMs, RT—R179 Tween 80 cultured *M*.*tb*, RNT—R179 non-Tween 80 (detergent free) cultured *M*.*tb*.

In order to assess whether *M*.*tb* cultured in the presence or absence of Tween 80 had a differential effect on the host response, analysis of the transcriptome through RNAseq was employed (Fig 1).

When comparing uninfected BMDMs (control) to BMDMs infected with R179NT *M*.*tb*, 3453 differentially expressed genes were identified. The comparison of BMDMs infected with R179T *M*.*tb* and BMDMs infected with R179NT *M*.*tb* revealed 2466 differentially expressed genes. Further, 2208 genes show no differential expression between these infection conditions (Fold change between -2 and 2 and with a FDR < 0.05).

Table 1 reflects the top 20 up- and downregulated genes expressed in BMDMs infected with detergent-free cultured *M*.*tb* (R179NT) vs. Tween cultured *M*.*tb* (R179T). Refer to S1, S2 and S3 Tables for the complete set of differentially expressed genes. RNA-seq data have been deposited in the NCBI Gene Expression Omnibus (GEO) database with experiment series accession number [GSE72003].

**Table 1:** Top 20 up- and downregulated transcripts in BMDMs infected with R179NT *M.tb* vs. R179T *M.tb*.

**Upregulated**			
**Gene symbol**	**P-value**	**FDR step up**	**Fold change (RNT vs. RT)**
***Csf3***	2.23E-06	3.86E-04	16147.13
***Orm1***	1.42E-06	3.27E-04	7310.51
***Slc32a1***	1.78E-09	9.97E-06	4601.55
***Il23a***	1.12E-06	3.21E-04	3874.65
***4933416M07Rik***	6.91E-06	6.28E-04	3401.04
***Draxin***	1.46E-03	1.08E-02	2716.99
***Lypd6b***	2.08E-06	3.69E-04	2531.70
***Il23r***	1.34E-06	3.25E-04	2341.40
***P3h2***	5.75E-07	2.82E-04	1828.57
***Il6***	1.71E-06	3.49E-04	1693.62
***Il1a***	1.89E-06	3.58E-04	999.73
***Cxcl3***	6.02E-07	2.82E-04	782.29
***Il12b***	1.90E-05	1.02E-03	720.08
***Il1b***	1.08E-06	3.21E-04	649.31
***Itgb8***	7.79E-03	3.25E-02	295.45
***Upp1***	1.32E-02	4.68E-02	284.23
***Slc1a2***	5.50E-03	2.58E-02	280.92
***Cxcl5***	1.41E-02	4.90E-02	256.37
***Serpinb2***	1.36E-04	2.85E-03	251.10
***Tcp10b***	8.45E-03	3.42E-02	190.95
**Downregulated**			
**Gene symbol**	**P-value**	**FDR step up**	**Fold change (RNT vs. RT)**
***Slco2b1***	7.13E-06	6.28E-04	-283.11
***Ung***	1.14E-06	3.21E-04	-138.02
***Tcf19***	7.27E-04	7.22E-03	-69.38
***Cables1***	3.67E-04	4.86E-03	-52.11
***Nptx1***	7.62E-03	3.21E-02	-51.42
***Rtn4rl1***	1.32E-05	8.46E-04	-47.55
***Epha2***	3.01E-06	4.52E-04	-46.69
***Exo1***	1.64E-04	3.15E-03	-46.21
***Uhrf1***	1.66E-03	1.17E-02	-44.22
***E2f7***	4.94E-03	2.40E-02	-42.21
***Cd207***	1.11E-03	9.20E-03	-41.91
***Gm4980***	1.41E-06	3.27E-04	-39.32
***Mybl2***	2.27E-04	3.77E-03	-38.62
***Dtl***	2.09E-06	3.69E-04	-37.67
***Rgs7bp***	2.78E-05	1.27E-03	-36.68
***Pcp4l1***	2.00E-03	1.32E-02	-32.96
***Hpgd***	1.40E-03	1.06E-02	-32.22
***Ccne2***	2.17E-04	3.69E-03	-31.69
***Gm5086***	5.75E-04	6.30E-03	-31.30
***Nanos1***	1.43E-04	2.93E-03	-30.89
***Rrm2***	1.06E-05	7.63E-04	-30.56

Gene ontology enrichment analysis using IPA indicated that the top canonical pathways activated by BMDMs infected with detergent-free R179 *M*.*tb* were associated primarily with the DNA damage response, cell cycle control and pattern recognition receptors involved in the recognition of bacteria and viruses (Table 2). Interestingly, the aryl hydrocarbon receptor (AHR) signaling pathway is upregulated in the host cell. Recently, the role of this pathway in modulating the immune response was recently evaluated in BCG [27], however the role of AHR in *M*.*tb* infection is still largely unknown. Activation of the TREM1 pathway is a significant finding since it was the top canonical pathway activated in the blood of patients with active TB (analysis of eight independent genome wide expression studies [28]).

**Table 2:** Top Canonical Pathways activated in BMDMs after infection with detergent-free *M*.*tb*.

Pathway	P-value	Overlap (%)
Hereditary Breast Cancer Signaling	6.25E-13	38.1 (48/121)
Cell cycle control of chromosomal replication	2.91E-12	73.1 (19/26)
Role of BRCA1 in DNA Damage Response	3.27E-12	44.9 (35/78)
Aryl Hydrocarbon Receptor Signaling	2.44E-11	35.3 (47/133)
Mismatch Repair in Eukaryotes	2.99E-11	87.5 (14/16)
Role of Pattern Recognition Receptors in Recognition of Bacteria and Viruses	1.14E-10	36.2 (42/116)
ATM Signaling	5.62E-10	45.8 (27/59)
Small Lung Cancer Signalling	3.45E-09	40.8 (29/71)
TREM1 Signaling	7.63E-09	40.6 (28/69)

The analysis of upstream regulators (Table 3) indicate that Ptger4, a receptor which associates with prostaglandin E2 is inhibited. By inhibiting this receptor, host-cell death by necrosis is predicted [29]. TICAM-1 which is an adaptor molecule that participates in Toll-like receptor 3–mediated interferon-β induction [30] is increasingly activated in BMDMs infected with detergent-free cultured *M*.*tb*. As expected, both Ifng and Tlr4 are both predicted to be activated.

**Table 3:** Top Upstream regulators activated in BMDMs after infection with detergent-free *M*.*tb*.

Upstream Regulator	p-value of overlap	Predicted Activation
PTGER4	2.79E-46	Inhibited
TICAM1	5.63E-40	Activated
CSF2	8.85E-40	Inhibited
IFNG	1.39E-37	Activated
TLR4	1.46E-35	Activated

### Detergent-free cultured *M*.*tb* induce different host receptor gene transcription profiles

**Fig 2:**
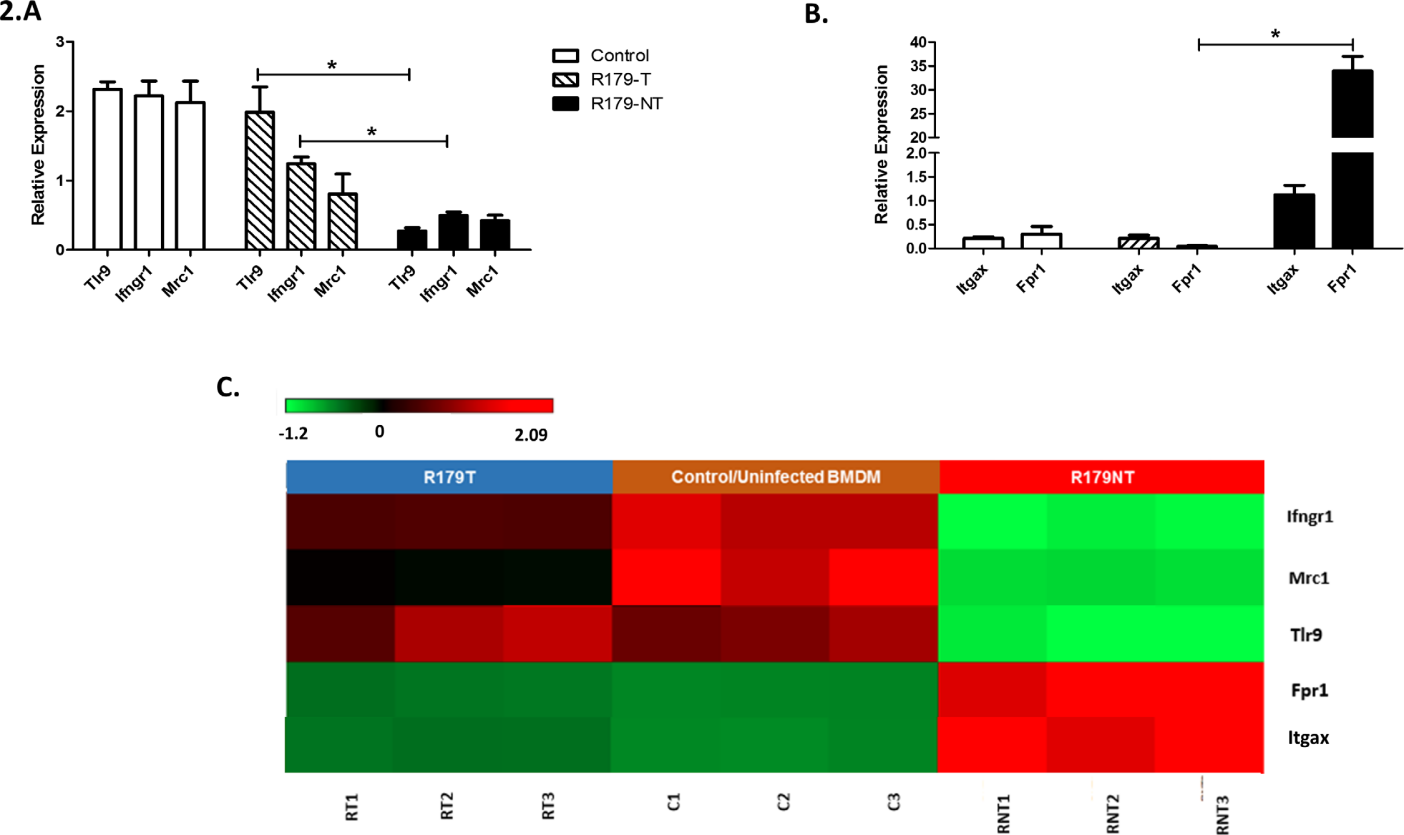
qPCR based validation of selected differentially expressed host receptor genes. **A.** Relative expression (fold change) of downregulated receptors in BMDMs infected with R179T and R179NT *M*.*tb*. **B.** Relative expression (fold change) of upregulated receptors BMDMs infected with R179T and R179NT *M*.*tb*. The means and standard error of three independent experiments are shown, * indicates significance *p* < 0.05. Legend corresponds to both graphs (A and B).**C.** Corresponding heatmap visualization of differentially expressed transcripts as analyzed by RNA-seq. The level of expression of each gene, in each sample, relative to the mean level of expression of that gene across all of the samples, is represented by using a red–green color scale as shown in the key with a range of − 1.2 to + 2.09 on a log(10) scale. Red = upregulation, green = downregulation (FC >2.0 and FDR <0.05).

To confirm the RNAseq results, we analysed a number of differentially expressed genes involved in *M*.*tb*-induced host receptor responses (Fig 2A and 2B). A concordance of 58% and a *r*-value of 0.76 (*p*-value < 0.01) was obtained across all genes selected for biological validation by qPCR (i.e. RNA extracted in a separate experiment). Interestingly, infection-induced transcriptional responses of both the mannose receptor (MRC1) and CD11c (Itgax) receptor are similar when challenged with both Tween-cultured and detergent-free *M*.*tb*, which suggests that the presence of Tween does not significantly affect the mycobacterial ligands which associate with these receptors. Macrophages infected with R179NT *M*.*tb* exhibit significantly lower transcript levels of Ifngr1 and Tlr9 in comparison to uninfected macrophages (Fig 2A). *Ifngr1* and *Tlr9* show significant differences between macrophages infected with R179T and R179NT *M*.*tb*. The indirect association of these two receptors in highlighted by the fact that the downregulation of Tlr9 suppresses the release of IFNα [31] and therefore Ifnγ [32], which is important for the successful clearance of *M*.*tb*. Through the downregulation of Ifngr1, it may be suggested that the host cell responds weakly to Ifnγ, thereby increasing the chance of the intracellular survival of M.tb. Fpr1 expression is highly stimulated upon infection with R179NT *M*.*tb*, which is not observed in response to infection with R179T *M*.*tb* (Fig 2B). This receptor is stimulated by the presence of bacterial formyl peptides but also by endogenous ligands such as annexin 1 [33] and mitochondrial formylated peptides (only released upon mitochondrial lysis [34]). Since these are both associated with conditions of cellular stress, it may suggest that infection with detergent-free *M*.*tb* is perceived by the host cell as potentially threatening, a response which is not induced by cells infected with Tween-cultured *M*.*tb*.

### Detergent-free cultured *M*.*tb* elicit a robust pro-inflammatory transcriptional response in BMDMs

**Fig 3:**
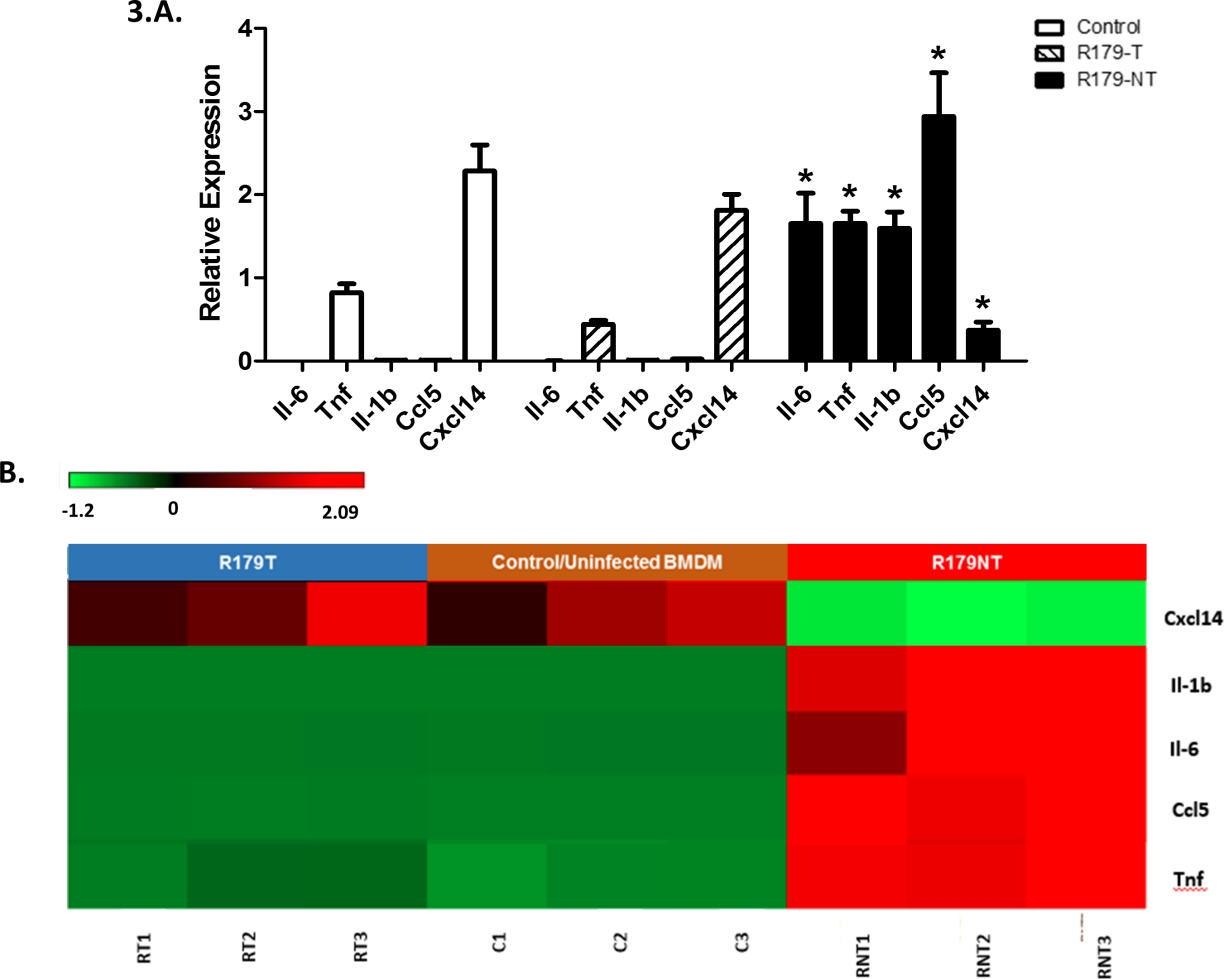
qPCR based validation of selected differentially expressed host cytokine and chemokine genes. **A.** Relative expression (fold change) of cytokines and chemokines in BMDMs infected with R179T and R179NT *M*.*tb*. The means and standard error of three independent experiments are shown, * indicates significance p < 0.05 vs. R179T. **B.** Corresponding heatmap visualization of differentially expressed transcripts as analyzed by RNA-seq The level of expression of each gene, in each sample, relative to the mean level of expression of that gene across all of the samples, is represented by using a red–green color scale as shown in the key with a range of − 1.2 to + 2.09 on a log(10) scale, Red = upregulation, green = downregulation (FC >2.0 and FDR <0.05).

Pro-inflammatory cytokine expression was then analysed (Fig 3A) to determine whether downstream responses were affected. *Il-6*, *Tnf*, *Il-1b and Ccl5* were induced in response to R179NT *M*.*tb*. Cxcl14 was significantly downregulated by R179NT *M*.*tb* which was not observed in BMDMs infected with R179T *M*.*tb*. Interestingly, the cytokine profile observed in R179T BMDMs is similar to that of the uninfected macrophage which is also displayed by the heat map generated for the selected set of genes (Fig 3B).

**Fig 4:**
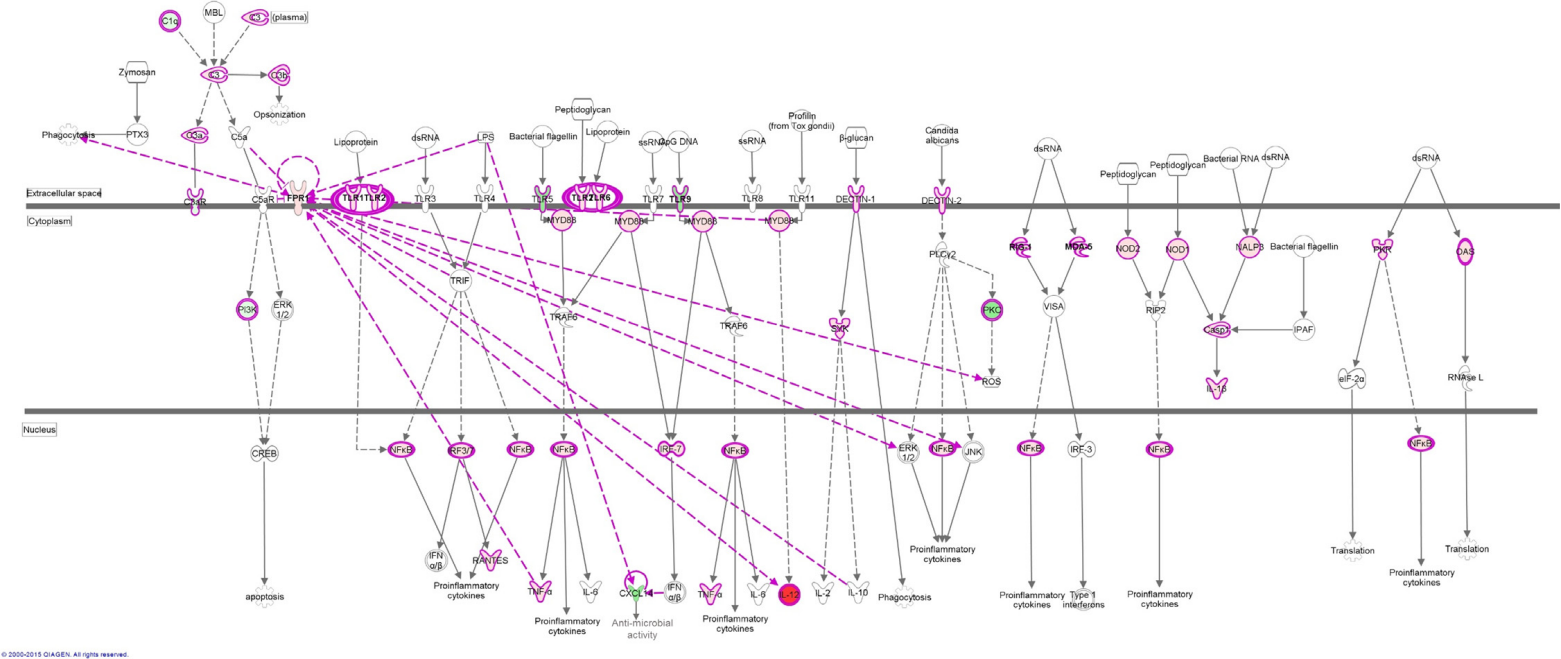
The host response to infection with detergent-free cultured *M*.*tb*. Using the canonical pathway from IPA depicting the ‘Role of Pattern Recognition Receptors in Recognition of Bacteria and Viruses’, expression values from RNAseq data was overlaid where red molecules indicate upregulation and green molecules indicate downregulation. Uncoloured molecules indicate no expression. Tlr1, 2 and 6 are stimulated by *M*.*tb* infection, whereas Tlr5 and 9 expression is downregulated. We included Fpr1, as it is highly stimulated upon infection with R179NT *M*.*tb*. Under infection conditions, it indirectly activates a number of proinflammatory cytokines as well as ERK1/2 and JNK. Cxcl14 was also included in the pathway as it was observed to be downregulated by *M*.*tb* infection. It has anti-microbial properties and was observed that infection by other pathogens decreases its expression. Solid lines indicate direct activation, broken lines indicate indirect activation.

Using IPA, we utilized the ‘Role of Pattern Recognition Receptors in Recognition of Bacteria and Viruses’ canonical pathway to overlay values from our data set (Fig 4). This provides a visual representation of the host cell responseto detergent-free cultured *M*.*tb*. Using the ‘Build’ and ‘Connect’ tools, Fpr1 and Cxcl14 were included into the pathway (their associations with other molecules are presented with pink lines). Interestingly we observe a possible role for Tlr5 in response to detergent-free *M*.*tb*. Tlr5, which associates with bacterial flagellin and activates Tnfα and NF-ĸB [35] is downregulated in host cells infected with detergent-free *M*.*tb*. Taken together, the above results suggest that by omitting Tween 80 from culture medium, *M*.*tb* interacts with the host in a slightly different manner and prompts further studies to fully characterise this response

## Discussion

The *Mycobacterium tuberculosis* cell wall components are essential for associating with macrophage cell surface receptors in order for effective internalisation and innate immune response initiation. Studies focussing on *M*.*tb* and other pathogens suggest that a bacterial cell wall in its native form results in a largely differential host-response to infection [4, 24, 36–39]. Tween 80 and other detergents solubilize membranous lipids and proteins on the *M*.*tb* cell wall [40] and affect macrophage uptake and subsequent innate immune response activation [3]. Since aerosolized *M*.*tb* enter the host in an unaltered, native state (i.e. detergent-free), it is important to assess the host response to such bacteria *in vitro*.

A clinical MDR strain (R179 *M*.*tb*), which was found to be a major contributor to an outbreak of drug-resistant tuberculosis in the Western Cape region of South Africa was used [25, 26] and cultured in detergent-free growth media (as described in Materials and Methods). The global host response to *M*.*tb* through RNAseq analysis strongly suggests that by omitting Tween 80 from the growth medium, *M*.*tb* elicits a largely differential response in the host cell (Fig 1). IPA was used to assess the top canonical pathways activated in the host in response to detergent-free cultured *M*.*tb* (Table 2). Of interest was the activation of the aryl hydrocarbon receptor pathway and TREM1 signaling. Notably, the activation of TREM1 signaling has been observed in active TB cases [28]. TREM1 also cooperates with TLR4 as a receptor complex [41] and therefore may be active in the innate immune response. As mentioned previously, the role of AHR pathway has been characterized in response to infection with BCG [27], however our results provide additional motivation for further investigation into this pathway.

Since Tween 80 solubilize membranous lipids and proteins on the *M*.*tb* cell wall [40] we selected various genes encoding host receptors which are known to associate with these receptors, as well as receptors that were observed to only be stimulated by infection with detergent-free cultured *M*.*tb*, to be evaluated through qPCR (Fig 2A and 2B). Recently, the role of *Tlr9* in host resistance to *M*.*tb* suggests an important role in the proinflammatory state, however its role in inducing the Th1 response is still confounding [42–44]. Functionality studies employ Tlr9 knock-out mice to assess the role of this receptor in inducing pro-inflammatory cytokines such as TNFα and Ifnγ, but data for direct measurement of transcription levels of Tlr9 mRNA in response to *M*.*tb* infection is not yet available. It is suggested that the downregulation of Tlr9 suppresses the release of Ifnα [31] and therefore Ifnγ [32], which is important for the successful clearance of *M*.*tb*. Here we present evidence that the host downregulates Tlr9 gene expression after infection with R179NT *M*.*tb*, which is not observed in host cells infected with *M*.*tb* cultured in the presence of Tween 80. This is the first direct assessment of *Tlr9* transcriptional changes associated with *M*.*tb* host cell infection. Due to the nature of the association observed between Tlr9 and Ifnγ (addressed above), we assessed the mRNA expression of its receptor IfnγR1. Interestingly, only *M*.*tb* cultured in the absence of detergent induced a downregulation in Ifngr1 expression. Our results agree with others as various infections, such as *Leishmania donovani* [45], *Trypanosoma cruzi* [46], and *Mycobacterium avium* [47] have been shown to downregulate Ifngr1expression. It is suggested that the inability of infected cells to respond to IFNγ, due to the downregulation of Ifngr1 expression results in the survival and persistence of *M*. *tuberculosis* in the infected host [48]. This suggests that detergent-free cultured *M*.*tb* may possess virulent properties which are absent in *M*.*tb* cultured in Tween.

Another receptor differentially expressed receptor was formyl peptide receptor 1 (FPR1). Signalling through Fpr1 induces inflammation, chemotaxis and phagocytosis, and is recognized by mycobacteria reactive T cells [49]. In this study, BMDMs infected with R179NT *M*.*tb* induced significant upregulation of *Fpr1* (Fig 2A and 2B), which is in agreement with a number of human studies [50–52]. Interestingly, the expression of this receptor under *M*.*tb*-infected conditions in *in vitro* and *in vivo* is not as pronounced, and may be due to the inability of Tween-cultured *M*.*tb* to stimulate this receptor. Our results suggest that at the host-pathogen interface, the interaction of such host receptors are largely variable between BMDMs infected with both Tween 80 and detergent-free cultured *M*.*tb*. We therefore hypothesized that the downstream signalling response should reflect this.

Pro-inflammatory cytokine and chemokine production is enhanced almost immediately after stimulation of the host receptors. An earlier study by Sani and colleagues assessed the effects on Tween use on the proinflammatory cytokine response of the host cell [4] and observed a differential proinflammatory response to infection with detergent-free cultured *M*. *bovis* BCG. In this study, the cytokine/chemokine profile exhibits a far more robust response to infection with detergent-free *M*.*tb* (Fig 3A and 3B). We observed that BMDMs infected with detergent-free *M*.*tb* induced a significant decrease in Cxcl14 mRNA, which was not observed by BMDMs infected with Tween-cultured *M*.*tb*. Cxcl14 is a chemokine that is constitutively expressed in normal tissues, however its receptor selectivity still remains largely unclear. Several reports indicate that it may play an anti-cancer role [53–55]. Additionally, it is suggested to have broad anti-microbial activity [56, 57] and is down-regulated by virulent pathogens in order to create a protected ecological niche during infection [58]. We present evidence for a role of Cxcl14 under TB-infection conditions which was previously unknown until now and provide necessary evidence for further exploration into its activity during TB infection.

In Fig 4 we attempt to summarise the host response to infection with detergent-free *M*.*tb* by using the IPA canonical pathway ‘pattern recognition receptors involved in the recognition of bacteria and viruses’. We have indicated roles for Fpr1 and Cxcl14 in this response and have additionally observed a possible role for Tlr5 during host infection with *M*.*tb*. Taken together our results indicate the observed host response to detergent-free *M*.*tb* suggests that this close to native state is perceived differently by the host cell. Additionally, host components involved in *M*.*tb* clearance such as Tlr9, Fpr1 and Cxcl14 are poorly initiated by host cells infected with *M*.*tb* cultured in detergent.

We therefore suggest that future studies further elucidate the host response to infection to such bacteria. Additionally, detergents such as Tween 80 are not entirely suitable for culturing *M*.*tb* for use in infection experiments as this fails to provide a complete profile of infection-related events. Results should therefore be interpreted with caution.

## Supporting Information

S1 Fig R179 at different stages of the SSF method in preparation for infection of macrophages. Cultures were grown to an OD_600_ of 0.4 before making stocks, as described in Methods. A. Thawed stock vials; note how the detergent-free grown bacteria has completely settled out, while the Tween 80 grown bacteria is a homogenous suspension. B. ZN slide of stock bacteria after pipetting 10X with 1ml tip. Clumps are generally larger and the bacteria tightly packed for the detergent-free stock. C. After 10X syringing through 25G needle. D. The top 750 μl after 10min settling of major clumps. E. Bacteria in 5ml RPMI before filtration. G. Bacteria in 5ml after filtration through a 5.0 μm pore size filter.
(TIF)

S2 Fig ZN stains representing R179 *M*.*tb* grown up to different OD_600_ in detergent-free 7H9 after processing with the SSF method (in 5 ml host-cell growth medium). A. OD_600_ = 0.8 cultures generate fewer bacteria for infection experiments. B. OD_600_ = 0.4 cultures generate a higher concentration of bacteria for infection experiments (arrows indicate single-celled bacteria).
(TIF)

S3 Fig Intracellular *M*.*tb* represented through uptake measurements and ZN staining. **A**. BMDMs were infected with *M*.*tb* at a MOI 1–3. After 4 hours, BMDMs were lysed and CFUs plated out and the percentage uptake of R179-T and R179-NT was assessed. **B**. ZN stains of intracellular *M*.*tb* 4 hours after infection, 3 replicates are shown with 2 fields of view (F.O.V) each. Arrows indicate intracellular M.tb. Images were taken at 100x oil immersion.
(TIF)

S4 Fig Heatmap visualization and sample clustering of differentially expressed transcripts as analyzed by RNA-seq of the host response to BMDMs infected with R179NT (detergent-free vs. R179T (Tween 80). (TIF)

S1 Methods Partek settings. (DOCX)

S1 Table Differentially expressed genes in uninfected BMDMs vs. R179-NT *M*.*tb* (Detergent-free medium) infected BMDMs. (XLSX)

S2 Table Differentially expressed genes in uninfected BMDMs vs. R179-T *M*.*tb* (Tween 80 medium) infected BMDMs. (XLSX)

S3 Table Differentially expressed genes in BMDMs Infected with R179T *M*.*tb* vs. BMDMs Infected with R179NT *M*.*tb* (Tween 80 medium). (XLSX)

S4 Table Raw RNAseq data. (XLSX)
