# Supplementary figures and images for: The Host Response to a Clinical MDR Mycobacterial Strain Cultured in a Detergent-Free Environment: A Global Transcriptomics Approach
Source: PLoS One. 2016 Apr 7;11(4):e0153079. doi: 10.1371/journal.pone.0153079 (PMC4824497; doi:10.1371/journal.pone.0153079)

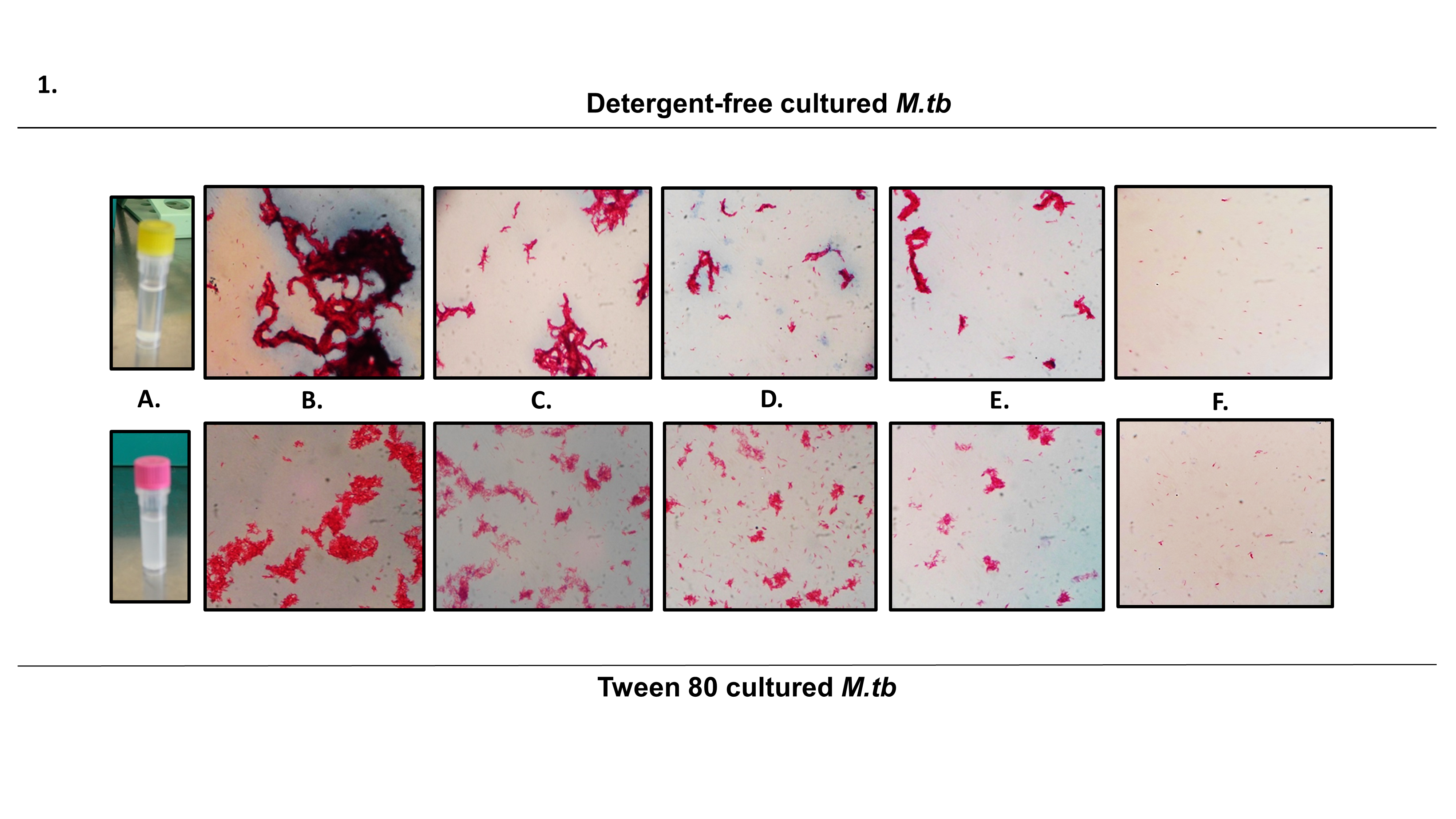

Supplement: S1 Fig — Cultures were grown to an OD600 of 0.4 before making stocks, as described in Methods. A. Thawed stock vials; note how the detergent-free grown bacteria has completely settled out, while the Tween 80 grown bacteria is a homogenous suspension. B. ZN slide of stock bacteria after pipetting 10X with 1ml tip. Clumps are generally larger and the bacteria tightly packed for the detergent-free stock. C. After 10X syringing through 25G needle. D. The top 750 μl after 10min settling of major clumps. E. Bacteria in 5ml RPMI before filtration. G. Bacteria in 5ml after filtration through a 5.0 μm pore size filter. (TIF) [file pone.0153079.s001.tif]

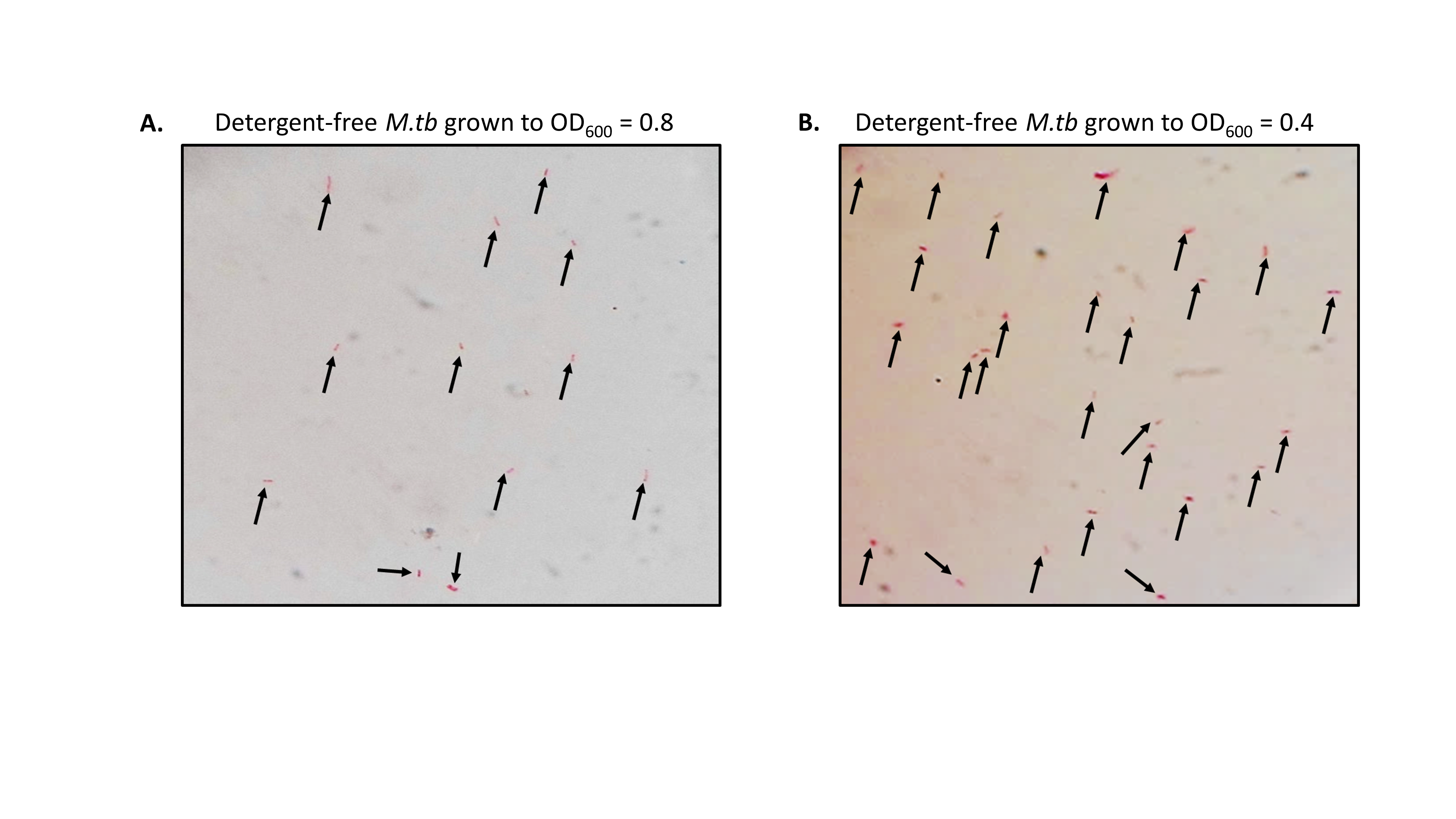

Supplement: S2 Fig — A. OD600 = 0.8 cultures generate fewer bacteria for infection experiments. B. OD600 = 0.4 cultures generate a higher concentration of bacteria for infection experiments (arrows indicate single-celled bacteria). (TIF) [file pone.0153079.s002.tif]

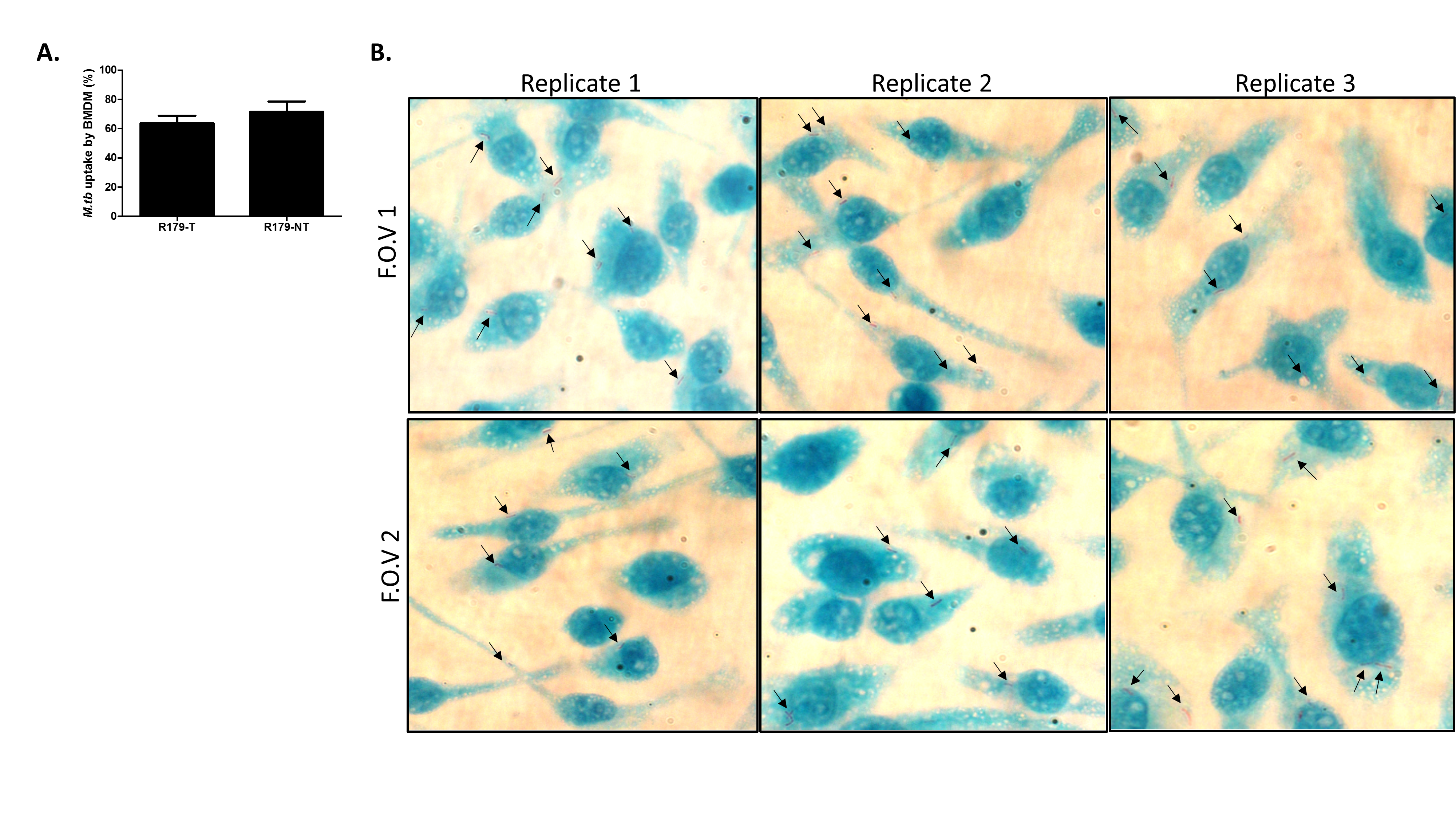

Supplement: S3 Fig — A. BMDMs were infected with M.tb at a MOI 1–3. After 4 hours, BMDMs were lysed and CFUs plated out and the percentage uptake of R179-T and R179-NT was assessed. B. ZN stains of intracellular M.tb 4 hours after infection, 3 replicates are shown with 2 fields of view (F.O.V) each. Arrows indicate intracellular M.tb. Images were taken at 100x oil immersion. (TIF) [file pone.0153079.s003.tif]

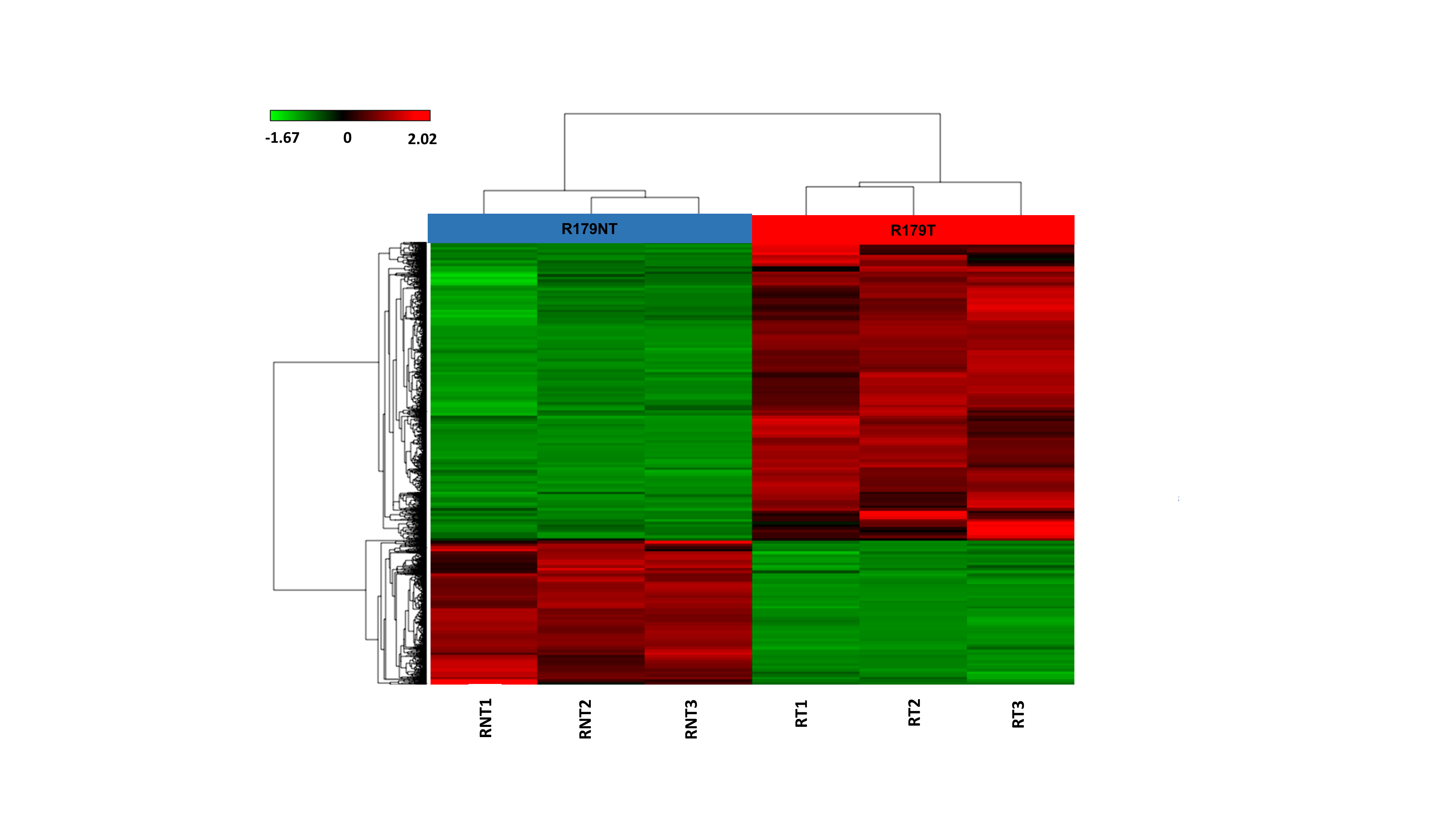

Supplement: S4 Fig — (TIF) [file pone.0153079.s004.tif]
