# Supplementary material for: The Host Response to a Clinical MDR Mycobacterial Strain Cultured in a Detergent-Free Environment: A Global Transcriptomics Approach
Source: PLoS One. 2016 Apr 7;11(4):e0153079. doi: 10.1371/journal.pone.0153079 (PMC4824497; doi:10.1371/journal.pone.0153079)
Supplement: S1 Methods — (DOCX) [file pone.0153079.s005.docx]

**S1 Methods**

**Partek Settings used.**

**Quantify to transcriptome (Partek E/M):**

Transcript model: mm10_refseq_15_02_02_v2.pannot

Input parameters

| **Option** | **Value** |
| --- | --- |
| Strict paired-end compatibility | true |
| Require junction reads to match introns | true |
| Strand specificity | No |
| Minimum read overlap with feature | 80 |
| Report unexplained regions | false |
| Include BAM files in output project file | false |

**Differential gene expression (GSA):**

Input parameters

| **Option** | **Value** |
| --- | --- |
| Run analysis on | Gene level |
| Filter type | Lowest maximum coverage --- 1 |
| Normalization offset | 1.0E-4 |
| Normalization type | RPKM (Default: Total Count) |
| FDR step-up | true |
| Storey q-value | false |
| Min error degrees of freedom | 0 |
| Model selection criterion | AICc |
| Enable multimodel approach | Yes |
| Use only reliable estimation results | Yes |
| P-value type | F (Default: Wald) |
| Normal | true |
| Lognormal | true |
| Negative binomial | true |

**Filters applied to GSA result:**

Total reads > 60

FDR <= 0.05

Fold change <-2 or >2 (exclude fold change between -2 and 2)
